# Supplementary material for: Baby makes three: Maternal, paternal, and zygotic genetic effects shape larval phenotypic evolution
Source: Evolution. 2021 Jun 7;75(7):1607–18. doi: 10.1111/evo.14244 (PMC8361925; doi:10.1111/evo.14244)
Supplement: Supplementary file 2 — Supplementary Material [file EVO-75-1607-s001.pdf]

G_2 Males

G_2 Females		X^LY		X^PY	
		X^L	Y	X^P	Y
X^LX^P	X^L	X^LX^L 2	X^LY 2	X^LX^P 1	X^LY 2
	X^P	X^LX^P 1	X^PY 0	X^PX^P 0	X^PY 0
X^PX^P	X^P	X^LX^P 1	X^PY 0	X^PX^P 0	X^PY 0
	X^P	X^LX^P 1	X^PY 0	X^PX^P 0	X^PY 0
